# Temporal examination of adult food insecurity amongst Canadian families managing food allergy

**DOI:** 10.1186/s13223-023-00802-6

**Published:** 2023-05-27

**Authors:** Michael A. Golding, Leslie E. Roos, Elissa M. Abrams, Jennifer D. Gerdts, Jennifer L. P. Protudjer

**Affiliations:** 1 Department of Pediatrics and Child Health, University of Manitoba, Winnipeg, MB Canada; 2 The Children’s Hospital Research Institute of Manitoba, 501G-715 McDermot Avenue, Winnipeg, MB R3E 3P4 Canada; 3 Department of Psychology, University of Manitoba, Winnipeg, MB Canada; 4 University of British Columbia, Vancouver, BC Canada; 5 Food Allergy Canada, Toronto, ON Canada; 6 Department of Food and Human Nutritional Sciences, University of Manitoba, Winnipeg, MB Canada; 7 George and Fay Yee Centre for Healthcare Innovation, Winnipeg, MB Canada; 8 Institute of Environmental Medicine, Karolinska Institutet, Stockholm, Sweden

**Keywords:** COVID-19, Food allergy, Food insecurity

## Abstract

**Background:**

Pediatric food allergy is associated with excess familial food costs compared to families without allergy. Since the start of the COVID-19 pandemic, food prices have increased substantially.

**Objective:**

To understand the temporal pattern of food insecurity amongst Canadian families with food allergy from the year prior to the pandemic, through May 2022.

**Methods:**

Using data collected electronically from families reporting food allergy using a validated food security questionnaire, we estimated food insecurity, including categories of food insecurity (marginal, moderate, secure) in the year prior to the pandemic (2019; Wave 1), and the first (2020; Wave 2) and second years of the pandemic (2022; Wave 3).

**Results:**

Participants in all waves were commonly in 2 + adult, 2 child households. Less than half of participants (Waves 1–3: 45.7%, 31.0%, and 22.9%, respectively) reported household incomes below the median Canadian. Common allergies were milk, eggs, peanuts and tree nuts. In Wave 1, 22.9% of families reported food insecurity; corresponding numbers at Waves 2 and 3 were 30.6% and 74.4%, respectively, representing an overall increase of 225.6%, including notable increases in severe food insecurity.

**Conclusion:**

Canadian families with pediatric food allergy report higher rates of food insecurity compared to the general Canadian population, especially during the pandemic.

**Supplementary Information:**

The online version contains supplementary material available at 10.1186/s13223-023-00802-6.

## Introduction

For families managing food allergy, dietary avoidance is essential to prevent a food allergic reaction [1], but has substantial financial costs [2, 3]. Food allergy is also associated with a psychosocial burden [4–6]. Previous research from our group dating prior to the COVID-19 pandemic demonstrates that Canadian families with food allergy face excess food costs associated with the purchase of allergy-friendly foods, in the order of magnitude of about $2400 Canadian per year, on average, or about $200 per month [2]. Moreover, we recently reported that, in the early months of the COVID-19 pandemic, families managing food allergy spent an additional $100—$200 per month on food, compared to immediately prior to the pandemic [7]. Canadian families overall have allocated a greater proportion of their household income to food purchases, with increases since the COVID-19 pandemic. Since 2019, grocery prices in Canada have increased due rates of inflation not seen since the early 2000s, and which have resulted in widespread concerns about food insecurity [8], defined as “the inability to acquire or consume an adequate diet or sufficient quantity of food in socially acceptable ways, or the uncertainty that one will be able to do so” [9]. But, for families managing food allergy and who already spend disproportionately more on food than families who do not manage food allergy, there is also a theoretically greater risk of food insecurity compared to families not managing food allergy.

With this in mind, we sought to understand the temporal pattern of food insecurity amongst Canadian families with food allergy from the year prior to the pandemic, through to May 2022.

## Methods

### Study populations and data sets

This study makes use of three distinct datasets, referred hereafter as waves. Wave 1 is based on data prior to the COVID-19 pandemic, collected between 14 and 18 April 2020, as part of the Parenting during the Pandemic study, in which parents of children ages 0–8 years were asked to report on rates of food insecurity in the 12 months prior to the pandemic. For Wave 1 data only, both families with food allergy and without food allergy were able to complete the questionnaire. However, the results presented herein are restricted only to those reporting a food allergy. Wave 2 data were collected in March 2020, immediately after the start of the global pandemic, to the end of 2020. Wave 3 data were collected in May 2022 (i.e., during Food Allergy Awareness Month). All data were collected via electronic platforms, in which participants responded to questions available online advertised widely through platforms such as Twitter, Facebook groups and Instagram at each of these 3 time points.

At all three waves, we collected data on food security (see below) and basic household demographic information. Post-secondary education was defined as a dichotomous variable (high school vs. at least some post-secondary education). Median income was defined as a binary cut-off of $70,000 Canadian dollars (CAD) at Wave 1, and $65,000 CAD for Waves 2 and 3, which approximates the median after-tax incomes for Canadian families in 2020 [10].

In the present analysis, our study population included participants who reported having at least one child with food allergy, and who completed the questions on food security. Participants themselves did not need to have food allergy.

### Food security

Both our data and the results presented by Statistics Canada [11, 12] used the Household Food Security Module of the Canadian Community Health Survey to assess food security (see Supplementary file for a further description of this module 1). In brief, this validated module has been used by the Government of Canada to monitor income-related food insecurity since 2005, and queries the financial ability to purchase balanced meals, as well as skipping meals or going hungry due to insufficient food or money to purchase food [12]. The module includes questions to assess food security amongst adults, and amongst children. Herein, we report on food insecurity amongst adults. Possible answers included never true, sometimes true, and often true; as well as no/yes. Affirmative answers included sometimes true, often true, yes, and were used to generate categories of food security or insecurity. Families were considered food secure if they had no affirmative responses. Amongst those reporting affirmative answers, marginal food insecurity was defined as one affirmative answer, moderate food insecurity was defined as two to four affirmative answers, and severe food insecurity reflected five or more affirmative answers.

Participants were asked to respond to the questions based on their situation in the year prior to the COVID-19 pandemic (Wave 1; recall period 12 months, from March 2019-March 2020), immediately after the start of the global pandemic, to the end of 2020 (Wave 2; recall period 9 months, from 11 March 2020 to 31 December 2020), and since March 2021 (i.e. during the second year of the pandemic; Wave 3; recall period 3–6 months, 11 March 2021–September 2021).

For Wave 1, we also reported the proportion of food insecurity amongst families without food allergy, as a comparison.

### Food allergy

Food allergy-specific questions included, during all waves, parent-reported types of food allergies (priority allergens in Canada [13]; other food allergies).

During Waves 2 and 3, we collected information on specific types of food allergy in addition to information on method of diagnosis, e.g. type of specialist, whether diagnosis was made by a specialist or general physician and whether or not the child carried an EAI.

### Statistical analysis

Data were described using parentheses (n/N, %), mean, and standard deviation (SD), using Stata 17.0 (College Station, TX). Data collection was approved by the University of Manitoba (Wave 1: Research Ethics Board 1; Waves 2 and 3: Health Research Ethics Board).

## Results

### Participant characteristics

At Wave 1, 35 participants who completed the questions on food security also reported having a child with food allergy (Table 1). By comparison, 49 participants were included in the final sample at Wave 2 and 39 in Wave 3. Participants in Wave 1 were commonly in 2 + adult households (85.7%), had post-secondary education (85.7%), and had an average of two children. About half of participants (16/35; 45.7%) reported household incomes ≤ $70,000 Canadian dollars (CAD). Across all three waves, Manitoba was the most common province of residency (Wave 1: 68.6%; Wave 2: 89.8%, Wave 3: 34.3%). The three most commonly reported food allergies were eggs (20.0%), peanuts (17.1%) and milk (17.1%). Multiple food allergies were common (Wave 1: 14.3%, Wave 2: 61.2%, Wave 3: 48.7%). EAI possession was reported in Wave 2 (67.3%) and Wave 3 (25.6%); no data were available for Wave 1. The characteristics of participants during Waves 2 and 3 were similar to those at Wave 1, with the exception of the three most common allergens (Wave 2: milk [47.9%], peanuts [47.9%] and tree nuts [36.7%]; Wave 3: peanuts [41.0%], milk [33.3%], tree nuts [20.5%]). As well, children in Wave 3 were, on average, 8.77 ± 5.98 years, whereas, in Wave 1, the maximum age was 8.99 years.

**Table 1:** Participant characteristics

	Wave 1 (N = 35)		Wave 2 (N = 49)		Wave 3 (N = 39)	
	n	%	n	%	n	%
Household demographics						
2 + adult household	30	85.7	45	91.8	25	64.1%
Mean (± SD) number of children	35	1.91 ± 0.82	48	1.81 ± 0.67	39	1.59 ± 0.72
Post-secondary education	30	85.7	37	88.1	25	73.5
Below median income	16	45.7	13	31.0	8	22.9
Manitoba residency*	24	68.6	44	89.8	12	34.3
Child characteristics†						
Aged < 18 months‡	13	62.9	–	–	–	–
Aged 18 months to 4 years‡	21	60.0	–	–	–	–
Aged 5 to 8 years‡	13	37.1	–	–	–	–
Mean (± SD) age of children‡	–	–	47	5.67 ± 4.69	20	8.77 ± 5.98
Food allergy characteristics						
At least two food allergies	5	14.3	30	61.2	19	48.7
Milk	6	17.1	23	47.9	13	33.3
Eggs	7	20.0	14	29.2	7	17.9
Peanuts	6	17.1	23	47.9	16	41.0
Tree nuts	2	5.7	18	36.7	8	20.5
Fish	2	5.7	6	12.5	4	10.3
Shellfish	0	0.0	4	8.3	4	10.3
Soy	1	2.9	4	8.3	4	10.3
Wheat	1	2.9	3	6.3	1	2.6
Sesame	0	0.0	–	–	4	10.3
Mustard	0	0.0	1	2.1	1	2.6
Other	3	8.6	9	18.4	4	10.3
EAI possession‡	–	–	33	67.3	10	25.6

*CAD* Canadian dollars, *EAI* epinephrine autoinjector, *SD* standard deviation

^*^For Wave 3, N = 35

^†^Not mutually exclusive

^‡^A dash (-) indicates that these variables were not queried for the respective wave

### Food insecurity amongst families managing food allergy

**Fig. 1:**
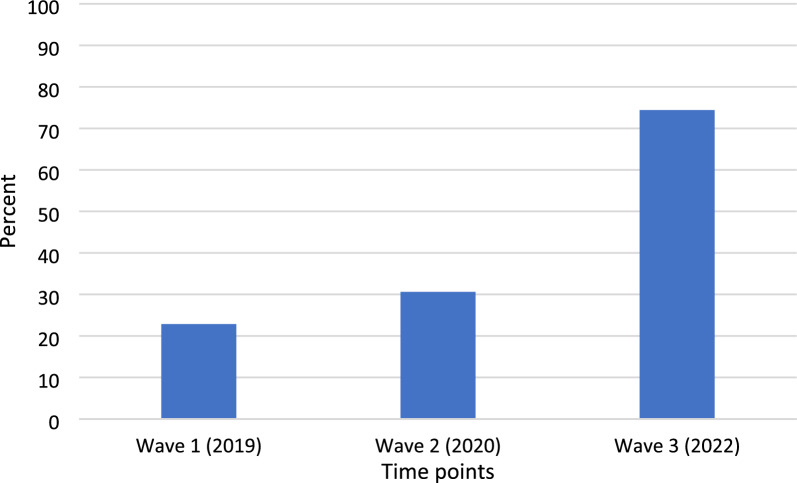
Prevalence of food insecurity amongst Canadian households managing food allergy between 2019 and 2022

In Wave 1, 22.9% of families managing food allergy reported food insecurity. Corresponding numbers for Waves 2 and 3 were 30.6% and 74.4%. (Figure 1)

**Fig. 2:**
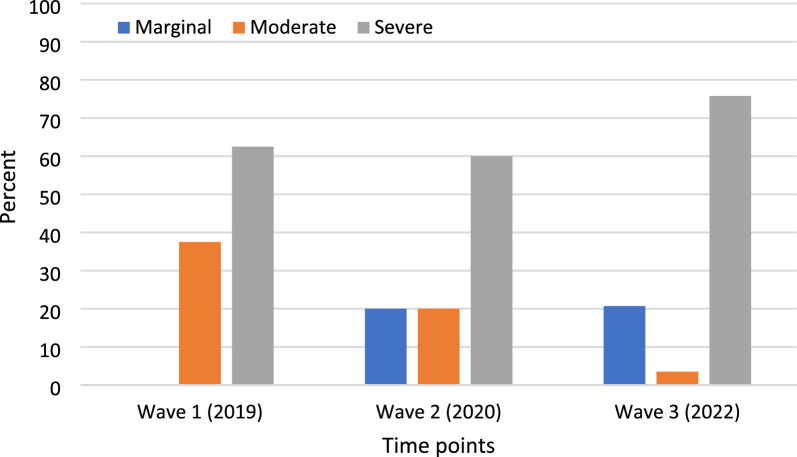
Category of food insecurity amongst Canadian households managing food allergy between 2019 and 2022

Amongst families managing food allergy and reporting food insecurity, the proportion experiencing moderate and severe food insecurity tended to decrease from Wave 1 to Wave 2, while marginal food insecurity appeared at Wave 2 only. Marginal food insecurity persisted to Wave 3, while the proportion of severe food insecurity increased substantially, with a corresponding decrease in the category of moderate food insecurity. (Figure 2)

### Food insecurity amongst families managing food allergy compared to the general population (Wave 1 only)

In Wave 1, 10.25% of participants without food allergy reported food insecurity.

## Discussion

This is the first analysis of food insecurity amongst Canadian families living with food allergy prior and during the COVID-19 pandemic. Across all three waves of this study, we identified an overall increase in the proportion of families with food allergy who also reported food insecurity, which more than doubled from 2019 to 2022. Moreover, food insecurity amongst those managing food allergy was two to 3 times greater, compared to the general Canadian population Compared to the proportion of food insecurity among the general Canadian population [11, 12], food insecurity was disproportionately higher amongst our study population. While food insecurity increased amongst those managing food allergy during Wave 1 to Wave 2, from 22.9% to 30.6%, respectively, the proportion of individuals with food insecurity in the general Canadian population remained stable over the same time period (10.6% and 9.6%, respectively).

Between 2019 and 2020, food prices increased by 2.7%, and increased again between 2020 and 2021 to 5% [14]. In 2022, food prices are predicted to continue to rise, by as much as 5–7% [8], an increase described as the “*largest increase in grocery prices in Canadian history*” [15]. Simultaneously, between 2021 and 2022, gasoline prices increased by more than 50% [16]. Unemployment rates have returned to seasonally-adjusted levels comparable to those reported prior to the pandemic [17] yet year-over-year wage growth remains low [18]. In brief, these factors collectively contribute to a near-perfect storm for increasing rates of food insecurity.

For families also managing food allergy, concerns regarding food insecurity are even greater, owing to the excess costs of food allergy, which both pre-dated [2] and has persisted during the pandemic [7]. Prior to the pandemic, these families spent, on average, $200 more per month on groceries, in comparison to families without food allergy [2]. These estimates were even higher one two months into the pandemic with high income families indicating that their spending on food had increased $200 per month in comparison to before the pandemic; whereas, lower income families spent additional $100 a month, on average [7]. The impact of these excess costs is seen in Waves 2 and 3, with an emergence of marginal food insecurity, and in Wave 3, with the near-disappearance of those reporting moderate food insecurity, and an increase amongst those reporting severe food insecurity.

At present, there are few resources across Canada to support families living with food allergy.

Since the year prior to the COVID-19 pandemic, demands for Canadian food banks have substantially increased, by an estimated 20.3%, across the country [19]. Many food banks report being unable to support families managing medical dietary requirements simply because of the increased demand for their services more broadly [20]. Recent data from the United States suggest that the majority of allergists do not regularly screen for food insecurity amongst their patient population and a smaller percentage report that they have not considered food insecurity amongst their patient population [21]. This is despite reports from many jurisdictions, including the United States [22], Canada [2] and Sweden [23] that families with children or adolescents living with food allergy face significantly higher costs, compared to their non-allergic controls.

We acknowledge the limitations of this study, including the small sample sizes from three different sources of data. The modest sample sizes found across the three waves may be a reflection of a general fatigue due to the acute switch to online services and learning as a result of the COVID-19 pandemic. As a result of the anonymous study design for all three waves, we have no way to confirm whether participants responded to the survey at multiple times. That said, all three waves made use of similar social media platforms for recruitment, and all used the same questionnaire on food insecurity. Nonetheless, our study population was largely composed of individuals with post-secondary education and reported household incomes above the national median. Moreover, a nationwide survey supports that Canadians in lower income households were less likely to report perceived food allergy [24]. A recent Canadian report supports that adults with lower levels of education are more vulnerable to experiencing food insecurity [25]. Taken collectively, the observed rate of food insecurity amongst adults with food allergy, in the present study, at 2 times greater amongst the general Canadian population, may, in fact, under-report food insecurity amongst those with food allergy. We also acknowledge that the majority of data were collected via online surveys, thus increasing the potential for a higher income population. We also lacked information on single vs. multiple food allergies in Wave 1. However, we believe that if anything, this recruitment method may similarly underrepresent the rates of food insecurity amongst these populations, rather than overrepresent them. That is, these rates presented herein may in fact be lower than the actual observed rate in the greater community. Long-term examinations of food insecurity amongst families managing food allergy must be conducted, particularly in light of high rates of inflation in Canada [26], and volatile situations internationally that may impact food supply chains [27]. Further stretching some families’ financial situations is the return or the repayment of the Canadian Emergency Response Benefit received during Wave 2 [28].

This study supports the role of healthcare professionals such as allergists and non-allergy physicians to screen for food insecurity amongst patients living with food allergy, and the role of policymakers to consider the needs of families facing medical dietary restrictions such as food allergy when developing programs and policies. Also warranting consideration is the number of families who must choose between refilling their EAI prescriptions and purchasing food, or fuelling their vehicles in order to facilitate, for example, a return to on-site work, due to an inability to afford all. A 2019 report estimated that approximately 1 million Canadians had to reduce household spending on food and heat to pay for medication, whereas one in five households reported a family member who could not fill a prescription in the previous year due to costs [29]. The implications of food prices on the ability to purchase medication since the start of the COVID-19 currently remains unclear. However, based on the increased costs of food, we speculate that the impact will be even greater than was reported in 2019. The findings from our study also highlight the need to consider the implications for social services and food banks and the organizations that fund them, including awareness of a need for supporting access to specialized food options (Fig. 2).

In conclusion, Canadian families living with food allergy face significantly higher rates of food insecurity compared to the general Canadian population. Since the year prior to the COVID-19 pandemic, rates of food insecurity have further increased in this population. Given the current rates of inflation and world events impacting food prices and food supply, these rates are expected, sadly, to increase.

## Supplementary Information

**Additional file 1.** Food security questions.

## Data Availability

Written requests for anonymous data will be considered by the authors.

## Abbreviations

CAD: Canadian dollars
EAI: Epinephrine autoinjector
SD: Standard deviation

## References

[1] Sicherer SH, Sampson HA (2018). Food allergy: a review and update on epidemiology, pathogenesis, diagnosis, prevention, and management. J Allergy Clin Immunol.

[2] Golding MA, Simons E, Abrams EM, Gerdts J, Protudjer JLP (2021). The excess costs of childhood food allergy on Canadian families: a cross-sectional study. Allergy Asthma Clin Immunol.

[3] Fong AT, Ahlstedt S, Golding MA, Protudjer JLP (2022). The economic burden of food allergy: what we know and what we need to learn. Curr Treat Options Allergy.

[4] Golding MA, Gunnarsson NV, Middeveld R, Ahlstedt S, Protudjer JLP (2021). A scoping review of the caregiver burden of pediatric food allergy. Ann Allergy Asthma Immunol.

[5] Westwell-Roper C, To S, Andjelic G, Lu Lin B, Soller L, Chan ES, Stewart SE (2022). Food-allergy-specific anxiety and distress in parents of children with food allergy: a systematic review. Pediatr Allergy Immunol..

[6] Acaster S, Gallop K, de Vries J, Marciniak A, Ryan R, Vereda A, Knibb R (2020). Psychosocial and productivity impact of caring for a child with peanut allergy. Allergy Asthma Clin Immunol.

[7] Golding MA, Lemoine-Courcelles C, Abrams EM, Ben-Shoshan M, Bégin P, Chan ES (2022). Changes in food-related costs during the COVID-19 pandemic among families managing food allergy. Front Allergy.

[8] Canada’s Food Price Report. 12^th^ edition. 2022. https://cdn.dal.ca/content/dam/dalhousie/pdf/sites/agri-food/Food%20Price%20Report%20-%20EN%202022.pdf 20220620.

[9] Government of Canada. Household food insecurity in Canada: overview. https://www.canada.ca/en/health-canada/services/food-nutrition/food-nutrition-surveillance/health-nutrition-surveys/canadian-community-health-survey-cchs/household-food-insecurity-canada-overview.html 20220620.

[10] Statistics Canada. Canadian Income Survey, 2020. https://www150.statcan.gc.ca/n1/daily-quotidien/220323/dq220323a-eng.htm 20220621.

[11] Caron N, Plunkett-Latimer J. Canadian income survey: food insecurity and unmet healthcare needs, 2018 and 2019. https://www150.statcan.gc.ca/n1/pub/75f0002m/75f0002m2021009-eng.htm 20220620.

[12] Polsky JY, Garriguet D. Household food insecurity in Canada early in the COVID-19 pandemic. https://www150.statcan.gc.ca/n1/pub/82-003-x/2022002/article/00002-eng.htm 20220620. 10.25318/82-003-x202200200002-eng 35179860

[13] Government of Canada. Allergens and gluten sources labelling. https://www.canada.ca/en/health-canada/services/food-allergies-intolerances/avoiding-allergens-food/allergen-labelling.html 20220621.

[14] Canada’s Food Price Report. 11^th^ edition. 2021. https://cdn.dal.ca/content/dam/dalhousie/pdf/sites/agri-food/Food%20Price%20Report%202021%20-%20EN%20(December%208).pdf 20220621.

[15] Charlebois S. Pandemic to cause largest increase in grocery prices in Canadian history: Sylvain Charlebois. https://retail-insider.com/retail-insider/2021/08/pandemic-to-cause-largest-increase-in-grocery-prices-in-canadian-history-sylvain-charlebois/ 20220621.

[16] Al Mallees N. As the cost of gas climbs, here’s what’s fueling the price at the pumps. https://www.cbc.ca/news/business/gas-prices-explained-2022-1.6460817 20220621.

[17] Statistics Canada. Labour force characterstics, monthly, seasonally adjusted and trend-cycles, last 5 months. https://www150.statcan.gc.ca/t1/tbl1/en/tv.action?pid=1410028701&pickMembers%5B0%5D=1.8&pickMembers%5B1%5D=3.1&pickMembers%5B2%5D=4.1&pickMembers%5B3%5D=5.1&cubeTimeFrame.startMonth=02&cubeTimeFrame.startYear=2020&cubeTimeFrame.endMonth=03&cubeTimeFrame.endYear=2022&referencePeriods=20200201%2C20220301 20220621.

[18] Statistics Canada. Labour force survey, March 2022. https://www150.statcan.gc.ca/n1/daily-quotidien/220408/dq220408a-eng.htm 20220414.

[19] Food Banks Canada. Canada’s food banks bracing as pandemic creates “perfect storm”—Food Banks Canada releases HungerCount 2021 Report. https://www.newswire.ca/news-releases/canada-s-food-banks-bracing-as-pandemic-creates-perfect-storm-food-banks-canada-releases-hungercount-2021-report-876497192.html 20220621

[20] Harvest Manitoba. COVID-19 is impacting Harvest’s food supply. https://www.harvestmanitoba.ca/neaed-food/need-food/ 20220621.

[21] Shroba J, Das R, Bilaver L, Vincent E, Brown E, Polk B (2022). Food insecurity in the food allergic population: a work group report of the AAAAI Adverse Reactions to Foods Committee. J Allergy Clin Immunol Pract.

[22] Gupta R, Holdford D, Bilaver L, Dyer A, Holl JL, Meltzer D (2013). The economic impact of childhood food allergy in the United States. JAMA Pediatr.

[23] Protudjer JLP, Jansson SA, Heibert Arnlind M, Bengtsson U, Kallström-Bengtsson I, Marklund B (2015). Household costs associated with objectively diagnosed allergy to staple foods in children and adolescents. J Allergy Clin Immunol Pract.

[24] Clarke AE, Elliott SJ, St-Pierre Y, Soller L, La Viellle S, Ben-Shoshan M (2021). Demographic characteristics associated with food allergy in a Nationwide Canadian Study. Allergy Asthma Clin Immunol.

[25] Polsky JY, Garriguet D. Household food insecurity in Canada early in the COVID-19 pandemic. Health Rep. 2022. https://www150.statcan.gc.ca/n1/pub/82-003-x/2022002/article/00002-eng.htm on 20230405. 10.25318/82-003-x202200200002-eng 35179860

[26] Statistics Canada. Consumer price index portal. https://www.statcan.gc.ca/en/subjects-start/prices_and_price_indexes/consumer_price_indexes on 20230406.

[27] Martin S. How war in Ukraine impacts food insecurity in Canada. The Independent. https://theindependent.ca/commentary/analysis/how-war-in-ukraine-impacts-food-insecurity-in-canada/ on 20230406.

[28] Government of Canada. Return or repay the Canadian Emergency Response Benefit (CERB). https://www.canada.ca/en/services/benefits/ei/cerb-application/return-or-repay.html on 20230406.

[29] Government of Canada. A prescription for Canada: achieving Pharmacare for all. https://www.canada.ca/en/health-canada/corporate/about-health-canada/public-engagement/external-advisory-bodies/implementation-national-pharmacare/final-report.html on 20220630.

