# Supplementary material for: Temporal examination of adult food insecurity amongst Canadian families managing food allergy
Source: Allergy Asthma Clin Immunol. 2023 May 27;19:45. doi: 10.1186/s13223-023-00802-6 (PMC10221737; doi:10.1186/s13223-023-00802-6)
Supplement: Supplementary file 1 — Additional file 1. Food security questions. [file 13223_2023_802_MOESM1_ESM.docx]

**Food Security Questions**

NOTES:

1. This questionnaire is based on the food security questions from the 2015 Canadian Community Health Survey. See: <https://www23.statcan.gc.ca/imdb/p3Instr.pl?Function=getInstrumentList&Item_Id=202664&UL=1V&>
2. As the present study only reported on adult food insecurity, we have limited this Supplement to include only questions on adult food insecurity, not child food insecurity.
3. For Wave 1, the questionnaire was written per the original language indicated by the CCHS. (i.e. In the past year…, with a slight modification: “in the 12 months prior to the COVID-19 pandemic…” This is the version we have presented in this supplement.
4. For Waves 2 and 3, the questionnaire was tailored to the COVID-19 pandemic.
   1. For Wave 2, the language was changed from “In the past year…” to “Since the start of the COVID-19 pandemic (declared by the World Health Organization on 11 March 2020….”
   2. For Wave 3, the language was changed from “In the past year…” to “since March 2021….”

**Questions**

**_____________________________________________________________________________________**

**Please think about your food situation since the start of the COVID-19 pandemic (declared by the World Health Organization on 11 March 2020). Each statement has three possible answers: often true, sometimes true or never true. Please select the answer that best reflects your family’s situation in the months since the start of the COVID-19 pandemic.**

1. Which of the following statements best describes the food eaten in your household since the start of the COVID-19 pandemic?
2. You and other household members always had enough of the kinds of foods you wanted to eat.
3. You and other household members had enough to eat, but not always the kinds of food you wanted.
4. Sometimes you and other household members did not have enough to eat.
5. Often you and other household members didn't have enough to eat.
6. You and other household members worried that food would run out before you got money to buy more. Was that often true, sometimes true, or never true since the start of the COVID-19 pandemic?
7. Often true
8. Sometimes true
9. Never true
10. The food that you and/or other household members bought just didn’t last, and there wasn’t any money to get more. Was that often true, sometimes true, or never true since the start of the COVID-19 pandemic?
11. Often true
12. Sometimes true
13. Never true
14. You and other household members couldn’t afford to eat balance meals. Was that often true, sometimes true, or never true since the start of the COVID-19 pandemic?
15. Often true
16. Sometimes true
17. Never true
18. Since the start of the COVID-19 pandemic, did you or other adults in your household ever cut the size of your meals or skip meals because there wasn’t enough money for food?
19. Yes
20. No (Go to Question 9)
21. Don’t know; refuse to answer

5b. How often did this happen?

1. Almost every month
2. Some months but not every month
3. Only 1 or 2 months
4. Don't know / refuse to answer
5. In the past 12 months, did you (personally) ever eat less than you felt you should because there wasn't enough money to buy food?
6. Yes
7. No
8. Don't know / refuse to answer
9. In the past 12 months, were you (personally) ever hungry but didn't eat because you couldn't afford enough food?
10. Yes
11. No
12. Don't know / refuse to answer
13. In the past 12 months, did you (personally) lose weight because you didn't have enough money for food?
14. Yes
15. No
16. Don't know / refuse to answer

**IF AFFIRMATIVE RESPONSE, continue to STAGE 3; otherwise, skip to end.**

1. Since the start of the COVID-19 pandemic, did you or other adults in your household ever not eat for a whole day because there wasn't enough money for food?
2. Yes
3. No (If children <18 years in the household, ask Question 13; otherwise skip to end.)
4. Don't know / refuse to answer

10b. How often did this happen?

1. Almost every month
2. Some months but not every month
3. Only 1 or 2 months
4. Don't know / refuse to answer

**Scoring and Categories of Food Insecurity _____________________________________________________________________________________**

| **Category of food insecurity** | **Score** | **Definition** |
| --- | --- | --- |
| Marginal | No more than one affirmative answer | Worry about running out of food and/or limited food selection due to a lack of money for food |
| Moderate | 2 to 5 affirmative answers | Compromise in quality and/or quantity of food due to a lack of money for food |
| Severe | 6 or more affirmative answers | Miss meals, reduce food intake, and, at the most extreme, go day(s) without food |
